# *In vitro* and *in vivo* anti-malarial activity of limonoids isolated from the residual seed biomass from *Carapa guianensis* (andiroba) oil production

**DOI:** 10.1186/1475-2875-13-317

**Published:** 2014-08-13

**Authors:** Tiago B Pereira, Luiz F Rocha e Silva, Rodrigo CN Amorim, Márcia RS Melo, Rita C Zacardi de Souza, Marcos N Eberlin, Emerson S Lima, Marne C Vasconcellos, Adrian M Pohlit

**Affiliations:** Laboratório de Princípios Ativos da Amazônia, Coordenação de Tecnologia e Inovação, Instituto Nacional de Pesquisas da Amazônia, Avenida André Araújo, 2936, Petrópolis, 69067-375 Manaus, Amazonas Brasil; Programa de Pós-graduação em Química, Universidade Federal do Amazonas, Avenida General Rodrigo Octávio, 6200, Coroado I, Campus Universitário, 69077-000 Manaus, Amazonas Brasil; Programa de Pós-graduação em Biotecnologia, Universidade Federal do Amazonas, Avenida General Rodrigo Octávio, 3000, Coroado I, Campus Universitário, 69077-000 Manaus, Amazonas Brasil; Escola Superior de Ciências da Saúde, Universidade Estadual do Amazonas, Avenida Carvalho Leal, 1777, Cachoeirinha, 69065-001 Manaus, Amazonas Brasil; Instituto de Química, Universidade Estadual de Campinas, Caixa Postal 6154, 13083-970 Campinas, São Paulo Brasil; Faculdade de Ciências Farmacêuticas, Universidade Federal do Amazonas, Rua Comendador Alexandre Amorim, 330, Aparecida, 69103-00 Manaus, Amazonas Brasil

**Keywords:** Malaria, 6α-acetoxyepoxyazadiradione, 6α-acetoxygedunin, Andirobin, 7-deacetoxy-7-oxogedunin, 6α-hydroxy-deacetylgedunin, *Plasmodium falciparum*, *Plasmodium berghei*, Human fibroblasts, Antiplasmodial

## Abstract

**Background:**

*Carapa guianensis* is a cultivable tree used by traditional health practitioners in the Amazon region to treat several diseases and particularly symptoms related to malaria. Abundant residual pressed seed material (RPSM) results as a by-product of carapa or andiroba oil production. The objective of this study was to evaluate the *in vitro* and *in vivo* anti-malarial activity and cytotoxicity of limonoids isolated from *C. guaianensis* RPSM.

**Methods:**

6α-acetoxyepoxyazadiradione (1), andirobin (2), 6α-acetoxygedunin (3) and 7-deacetoxy-7-oxogedunin (4) (all isolated from RPSM using extraction and chromatography techniques) and 6α-hydroxy-deacetylgedunin (5) (prepared from 3) were evaluated using the micro test on the multi-drug-resistant *Plasmodium falciparum* K1 strain. The efficacy of limonoids 3 and 4 was then evaluated orally and subcutaneously in BALB/c mice infected with chloroquine-sensitive *Plasmodium berghei* NK65 strain in the 4-day suppressive test.

**Results:**

*In vitro*, limonoids 1-5 exhibited median inhibition concentrations (IC_50_) of 20.7-5.0 μM, respectively. In general, these limonoids were not toxic to normal cells (MRC-5 human fibroblasts). *In vivo*, 3 was more active than 4. At oral doses of 50 and 100 mg/kg/day, 3 suppressed parasitaemia *versus* untreated controls by 40 and 66%, respectively, evidencing a clear dose–response.

**Conclusion:**

6α-acetoxygedunin is an abundant natural product present in *C. guianensis* residual seed materials that exhibits significant *in vivo* anti-malarial properties.

**Electronic supplementary material:**

The online version of this article (doi:10.1186/1475-2875-13-317) contains supplementary material, which is available to authorized users.

## Background

In 2010, there were an estimated 219 million cases of malaria infection resulting in 660,000 deaths worldwide [1]. Chloroquine-resistant (CQR) *Plasmodium falciparum* is now widespread and there are growing numbers of reports of CQR *Plasmodium vivax* worldwide. In recent years, artemisinin-based combined therapy (ACT) has been introduced as the first-line of treatment for *P. falciparum* and for the treatment of CQR *P. vivax*. However, reports of *P. falciparum* exhibiting resistance to artemisinin derivatives in four Southeast Asian countries, and resistance to ACT in a region of Cambodia are increasing the interest in lead compounds for the development of a new generation of anti-malarial drugs [1–4].

Natural products are the direct or indirect sources of most of the drugs introduced in the past 30 years [5]. Natural products from plants are a rich source of lead compounds for the development of new drugs against protozoan parasitic diseases such as malaria [6–9]. Quinine [10] and artemisinin are potent antimalarial natural products from plants. Further development gave rise to synthetic quinoline and artemisinin classes of antimalarials that form the basis of ACT. Today, artemisinin derivatives (*e.g.* sodium artesunate, arteether, dihydroartemisinin) and quinolines (*e.g.* chloroquine, primaquine) are the basis of malaria treatment worldwide. The Amazon region has a rich tradition of plant use for the treatment of malaria and a number of natural products have been isolated and semi-synthetic derivatives prepared exhibiting important *in vitro* and *in vivo* anti-malarial properties [11].

**Figure 1:**
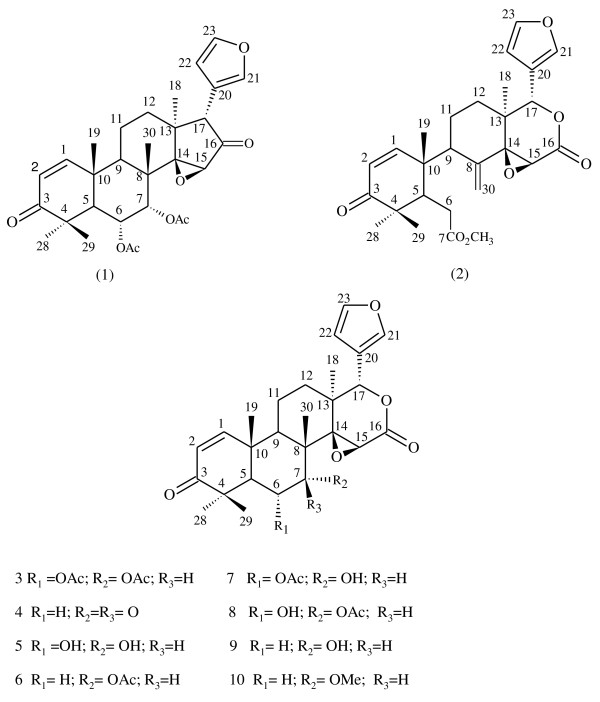
**Structures of limonoids isolated from** ***Carapa guianensis*** **and their semi-synthetic derivatives.** 6α-acetoxyepoxyazadiradione **(1)**, andirobin **(2)**, 6α-acetoxygedunin **(3)** and 7-deacetoxy-7-oxogedunin **(4)** were isolated herein from the residual seed material of *C. guianensis*. Semi-synthetic derivative 6α-hydroxydeacetylgedunin **(5)** was prepared herein. Gedunin **(6)**, andirolide H **(7)**, 6α-hydroxygedunin **(8)**, 7-deacetylgedunin **(9)** and 7-deacetyl-7-O-methylgedunin **(10)** were reported by others as indicated in the text*.*

Andiroba (*Carapa guianensis*) belongs to the Meliaceae family of plants. It is found in western India, South Africa and South America and is readily cultivated. Its trees can reach heights of 30 m and can produce 50 to 200 kg of seeds per year [12]. Andiroba oil, or carapa oil, as it is also known, is obtained from the seeds and has several uses in traditional medicine including the treatment of malaria [12–16]. A common method for obtaining andiroba oil begins by chopping the seeds into small pieces and then cooking on a fire-heated hot plate at a temperature of *cerca* 90°C. Then, the cooked seed meal is mechanically pressed to obtain the oil. This process generates a large quantity of residual seed material as a by-product that contains many bioactive constituents, including limonoids [13]. The following limonoids have been previously isolated from andiroba oil and are present in residual seed materials (Figure 1): 6α-acetoxyepoxyazaradione (**1**), andirobin (**2**), 6α-acetoxygedunin (**3**), 7-deacetoxy-7-oxogedunin (**4**), gedunin (**6**), 6α-hydroxygedunin (**8**), 1, 2-dihydro-3β-hydroxy-7-deacetoxy-7-oxogedunin, 17β-hydroxyazadiradione, methyl angolensate and xilocenin K [15, 17, 18].

Andiroba oil exhibits *in vitro* activity against *P. falciparum*. At concentrations of 8.2 μg/mL of the oil and 3.1 μg/mL of a limonoid-rich fraction obtained from this oil, 100% inhibition of the W2 strain of *P. falciparum* was observed after 24 h. Also, andiroba oil and a limonoid-rich fraction exhibited IC_50_ values of 9.4 and 2.4 μg/mL, respectively, after 48 h against the Dd2 strain of *P. falciparum* [19].

In a recent study, 16 limonoid components of the flowers of *Carapa guianensis* were isolated and nine of these components were tested for *in vitro* inhibitory activity against the FCR-3 strain of *P. falciparum*. Gedunin (**6**) and structurally-related limonoids **3**, **4**, **7** and **8** (Figure 1) exhibited the most *in vitro* inhibition (IC_50_ 2.5-2.8 μM) [20]. Despite the *in vitro* anti-plasmodial potential of gedunin and previous studies on its natural and semi-synthetic derivatives [20–24], *in vivo* anti-malarial data for gedunin-type limonoids in the literature are limited to gedunin itself and a semi-synthetic derivative of gedunin, 7-deacetyl-7*O*-methyl-gedunin (**10**) [22, 23]. These and other reasons prompted us to publish the present work on the *in vitro* anti-plasmodial activity of limonoids **1**-**4** isolated from *C. guianensis* and a semi-synthetic derivative **5** (Figure 1) and the *in vivo* anti-malarial activity of **3** and **4** in infected mice.

## Methods

Collection of andiroba seeds and production of residual pressed seed material (RPSM).

Collection took place on the morning of June 6, 2011. *Carapa guiananesis* seeds were collected at the National Institute for Amazon Research’s (INPA) Adolpho Ducke Forest Reserve located in Manaus, Amazonas State, Brazil from the areas beneath two trees identified by voucher specimens deposited previously in the INPA Herbarium under the accession numbers 192615 and 178658 [25]. Triage was performed by discarding perforated, marred and/or moldy seeds. In the municipality of Manaquiri, 64 km from Manaus, extraction of the oil was performed by first triturating the seeds (fresh weight 10 kg), heating (partially drying) the resulting ground seeds on a hot plate, followed by pressing the dried, ground seeds at room temperature in an industrial press. This last step led to the RPSM (5.3 kg) and crude andiroba oil (500 mL) containing suspended matter. After centrifuging, transparent, slightly yellow andiroba oil was obtained as an upper layer and the dark-coloured suspended matter (330 mg) was concentrated in the lower layer.

### Extraction and isolation

Residual pressed seed material (RPSM) (106.2 g) was continuously extracted (3 × 6 h) with acetone (300 mL) in a soxhlet apparatus. The combined acetone extracts were concentrated on a rotary evaporator and then freeze-dried. Dry acetone extract was dissolved in a mixture of 90:10 methanol/water (100 mL) and partitioned with hexanes (3 × 100 mL). The phases were separated and water was added to the methanol/water phase until a composition of 70:30 was reached. The latter was partitioned with chloroform (3 × 150 mL) and the combined chloroform fractions were totally evaporated. Column chromatography (CC) was performed on the combined chloroform fraction using silica gel 60 (particle size: 63-200 μm, column: d × h = 5.5 × 38.5 cm) and a gradient of hexanes and ethyl acetate (90:10, then 80:20), chloroform and ethyl acetate (90:10) and finally methanol (100%). The 40 chromatographic fractions obtained were analyzed by thin-layer chromatography (TLC) and combined into 12 fractions. Fraction 4 was further separated by CC on silica gel 60 (40-63 μm, d × h = 2.3 × 17 cm). Chloroform (100%) was used as eluent and 6α-acetoxyazadiradione (**1**) (105 mg) and andirobin (**2**) (24.2 mg) were obtained in pure fractions. Fraction 2 was further separated by CC on silica gel 60 (63-200 μm, d × h = 2.3 × 17 cm) and eluted with a gradient of hexanes/chloroform/ethyl acetate (56:33:11, 33:53:14, 20:50:30) to obtain 60 fractions that were combined into 33 fractions based on the similarity of their TLC profiles and 6α-acetoxygedunin (**3**) was obtained as a pure fraction (73.2 mg). A neighboring fraction was further purified by preparative TLC using hexanes/ether/butanol (82:9:9) which led to the isolation of more **3** (44.7 mg) and 7-deacetoxy-7-oxogedunin (**4**) (59.8 mg). Larger quantities of **3** and **4** for *in vivo* studies were available by extracting RPSM (1.1 kg) and liquid-liquid partitioning of the resulting extract (603.3 g) as described above followed by CC on the chloroform fraction (23.4 g) using silica gel 60 (40-63 μm, d × h = 5.5 × 40 cm) and eluted with hexanes/dichloromethane (40:60, then 20:80) and hexanes/dichloromethane/ethyl acetate (33:53:14, then 20:50:30), to yield a fraction containing **3** and **4** (1.9 g). CC on this fraction using silica gel 60 (40-63 μm, d × h = 4.3 × 30.5 cm) and elution with 97:3, 95:5 and then 90:10 mixtures of solvents A (95:5 hexanes/chloroform) and B (1:1 ether/butanol) yielded 3 combined fractions after TLC analysis. Fractions 1 and 3 contained pure **3** (325 mg) and **4** (395 mg), respectively. Fraction 2 (150 mg) was separated by preparative TLC using hexanes/ether/butanol (82:9:9) leading to isolation of more **3** and **4** (overall yields were 402 and 452 mg, respectively).

### Deacetylation of 3

This was carried out by dissolving **3** (40.5 mg, 75 μmol) in high performance liquid chromatography (HPLC) grade methanol (2.7 mL), adding anhydrous potassium carbonate (80 mg, 0.6 mmol) and magnetically stirring the resulting mixture at room temperature for 24 h. The reaction mixture was transferred using washings with chloroform (3 × 2 mL) and the resulting mixture was dried with saturated sodium chloride solution, then anhydrous sodium sulfate and then the drying agent was removed by filtration. The filtrate was totally evaporated and the resulting residue was recrystallized from HPLC grade methanol to obtain the diol product 6α-hydroxydeacetylgedunin (**5**) (12.8 mg, 28 μmol, 37%).

### Spectral analysis

High-resolution mass spectra (HRMS, Waters, Xevo Model) were obtained by direct infusion of each pure compound dissolved in methanol/water. Nuclear magnetic resonance (NMR) spectra were obtained of each pure sample on a Varian Inova 500 MHz and Bruker Avance, 600 MHz and 400 MHz. HRMS and full ^1^H and ^13^C NMR data on all isolated and prepared compounds are provided as Additional file 1.

### *In vitro*anti-plasmodial assay

*In vitro* cultures of the K1 strain of *P. falciparum* were established using A + type blood cells and Roswell Park Memorial Institute (RPMI 1640) culture medium enriched with 10% plasma in accordance with the Trager and Jensen procedure [26]. The *in vitro* inhibition of the growth of *P. falciparum* K1 from these cultures by limonoids **1**-**4** and derivative **5** was evaluated as described previously [27]. Briefly, this procedure involved preparing 5 mg/mL stock solutions of each compound in dimethyl sulfoxide (DMSO). The stock solutions were diluted to provide test solutions having concentrations in the range 0.13-100 μg/mL. Test solutions were transferred to 96-well test plates containing parasitized red blood cells with initial 2% haematocrit and 1% parasitaemia. Each sample was evaluated in duplicate and the test plate was incubated for 48 h at 37°C. After incubation, quantification of parasites was achieved by optical microscopy on blood smears from each well [27].

### Animals and ethical approval

Adult BALB/c mice (22 ± 3 g weight) were used for the *in vivo* anti-malarial tests and received water and food *ad libitum. In vivo* tests were performed using ‘Guidelines for Ethical Conduct in The Care and Use of Animals of the National Institute for Amazon Research (INPA)’. This work was authorized by INPA’s Commission of Ethics for the Use of Animals (CEUA 062/2012).

### *In vivo*anti-malarial assay

Limonoids **3** and **4** were assayed for *in vivo* anti-malarial activity in the Peters 4-day suppressive test against the *Plasmodium berghei* NK65 strain in BALB/c female mice, each inoculated with 1 × 10^5^ parasitized red blood cells [28]. The mice were randomly divided into groups of five individuals and individual groups were treated orally or subcutaneously with **3** or **4** at doses of 50 or 100 mg/kg/day over four days followed by evaluation of parasitaemia *versus* controls (negative control groups were treated with 2% DMSO vehicle and positive control groups with chloroquine) on days 5 and 7 following an established procedure [29]. Mortality was monitored in all groups for four weeks after inoculation. Two independent *in vivo* experiments were performed.

### Cytotoxicity assay

The MRC-5 cell line of human fibroblasts was grown in Dulbecco’s Modified Eagle’s Medium (DMEM) supplemented with 10% fetal bovine serum, 2 mM glutamine, 100 μg/mL streptomycin and 100 U/mL penicillin, and incubated at 37°C with a 5% atmosphere of CO_2_. For assays, the cells were plated in 96-well plates (2.5 × 10^4^ cells/well) and the AlamarBlue™ assay was performed using a previously described procedure [30, 31]. After incubation for 24 h, compounds **1**-**5** were individually dissolved in DMSO and the resulting solutions were diluted in culture medium. The resulting dilute solutions of each sample were added to wells at final (well) concentrations of 1.56-100 μg/mL. Control groups had final well concentrations of 0.1% DMSO. The plates were further incubated for 48 h. 3 h before the end of the incubation period, AlamarBlue™ (10 μL) was added to each well. The fluorescent signal was monitored with a multiplate reader using 530-560 nm excitation and 590 nm emission wavelengths.

## Results

Isolated and semi-synthetic limonoids **1**-**5** were fully characterized by high-resolution mass spectrometry (HRMS) and nuclear magnetic resonance (NMR) one and two-dimensional spectrometric techniques. Comparison of the NMR spectral data with literature data and analysis of the 1D and 2D NMR data allowed for the positive identification and spectral assignment of isolated limonoids **1** [32], **2** [33], **3** [34] and **4** [35] (Figure 1). Di-deacetylation of **3** led to the formation of a product that exhibited HRMS, ^1^H and ^13^C NMR data consistent with 6α-hydroxydeacetylgedunin (**5**). Compound **5** has been reported previously, however, no NMR data were provided [36].

Median inhibitory concentrations (IC_**50**_) of compounds **1**-**5** against *P. falciparum* K1 strain *in vitro* are summarized in Table 1. The IC_50_ values for these 5 compounds were in the range 5.0-20.7 μM. Semi-synthetic derivative **5** exhibited the most inhibitory activity. The *in vitro* anti-plasmodial activity of the other limonoids was moderate. The natural product **4** exhibited the least activity of all limonoids *in vitro*. Compounds **1**-**5** were not considered toxic to MRC-5 cells (**4** exhibited an IC_50_ = 47.3 μg/mL, all other IC_50_ values were > 100 μg/mL) over a period of 48 h.

**Table 1:** ***In vitro*** **IC**_**50**_ **values for limonoids from** ***Carapa guianensis*** **against** ***Plasmodium falciparum*** **and human fibroblasts**

		***Plasmodium falciparum***K1		Human fibroblasts MRC-5	
Limonoids	MW	IC _50_ (μM)	Result	IC _50_ (μM)	SI
6α-acetoxyazadiradione **(1)**	524	15.4	MA	>191	>12
andirobin **(2)**	492	15.3	MA	>203	>13
6α-acetoxygedunin **(3)**	540	7.0	MA	>185	>26
7-deacetyl-7-oxogedunin **(4)**	438	20.7	I	108 (87.2–134)	5
6α-hydroxydeacetylgedunin **(5)**	456	5.0	A	>219	>44
chloroquine diphosphate	516	0.33	A		
quinine sulfate	783	0.30	A		

Notes: IC_50_ ≤ 0.1 μM = highly active; 0.1 < IC_50_ ≤ 5 μM = active (A); 5 < IC_50_ ≤ 20 μM = moderately active (MA); IC_50_ > 20 μM = inactive (I). Selectivity index (SI) = IC_50_ (human fibroblasts)/IC_50_ (*P. falciparum*).

6α-acetoxygedunin (**3**) and 7-deacetoxy-7-oxogedunin (**4**) were each isolated on several hundred milligram scales which permitted further evaluation of these compounds in a rodent malaria model. The results of the suppressive test against *P. berghei* NK65 in infected mice are presented in Table 2. In general, limonoid **3** exhibited greater *in vivo* activity than **4**. The greatest *in vivo* activity (65.7% suppression of parasitaemia as compared to untreated controls) was observed for **3** administered orally at doses of 100 mg/kg/day. At a given dose and 5 or 7 days after infection, oral administration of **3** in general led to greater parasite suppression than did subcutaneous injection of this compound. Both oral and subcutaneous administration of **4** led to a suppression of parasitaemia in a clear dose–response. Mice that received **3** exhibited the longest survival times (24 ± 3 days) at oral doses of 100 mg/kg/day, however, these data were not statistically significant (T-test p > 0.05 compared to controls).

**Table 2:** ***In vivo*** **suppression of** ***Plasmodium berghei*** **NK65 in mice by limonoids isolated from** ***Carapa guianensis***

Dose (mg/kg/day)	% Parasite inhibition				Average survival time ± SD (day)	
	Oral		Subcutaneous		Oral	Subcutaneous
	5th day	7th day	5th day	7th day		
6α-acetoxygedunin **(3)**						
100	65.7	46.3	44.2	30.4	24 ± 3	22 ± 4
50	40.2	34.7	34.4	16.8	20 ± 3	21 ± 2
7-deacetoxy-7-oxogedunin **(4)**						
100	40.3	28.9	38.6	21.7	21 ± 3	22 ± 2
50	19.3	0	29.2	0	20 ± 2	21 ± 4
chloroquine diphosphate						
5	100	98	99	99	>40	>40
control	0	0	0	0	21 ± 2	21 ± 3

## Discussion

Gedunin (**6**) has been isolated previously from different parts of *Carapa guianensis* and exhibits *in vitro* activity (IC_50_ = 0.40–2.5 μM) against W2, D6 and FCR-3 strains of *P. falciparum* [20–22]. However, gedunin (**6**) was not isolated in the present work from RPSM. Interestingly, a recent study on the fruit from the same tree specimens from which fruit for the present study was collected described the isolation of 7-deacetyl gedunin (**9**), but not gedunin (**6**) [35]. Gedunin was considered to be an optimal structure for its high *in vitro* activity against different strains of *P. falciparum* and because structural modification of gedunin through semi-synthetic reactions yielded derivatives exhibiting decreased *in vitro* anti-plasmodial activity compared to gedunin [21, 22]. This previous work contributed to a partial understanding of structure-activity relationships for the *in vitro* anti-plasmodial activity of simple gedunin derivatives.

According to previous reports, deacetylation of gedunin (**6**) produced compound **9** that exhibited about 65 times less *in vitro* activity (IC_50_ = 2.6 and 1.3 μg/mL against D6 and W2, respectively) than gedunin [21] and the *in vitro* activity of 7-*O*-methyl-deacetylgedunin (**10**) and deacetylgedunin (**9**) are reported to be similar [22]. Based on these and other findings, it was presumed that the presence of a 7α-acetoxy moiety in the gedunin skeleton was important for *in vitro* anti-plasmodial activity.

Recently, the *in vitro* activity of limonoids **3** (IC_50_ = 2.8 μM) and **7** (IC_50_ = 4.0 μM) was reported against the FCR-3 strain of *P. falciparum* [20]. Both of these compounds exhibit a 6α-acetoxy moiety (however, only **3** exhibits a 7α-acetoxy moiety). Thus, a 7α-acetoxy group was not required for significant *in vitro* anti-plasmodial activity against *P. falciparum* as was thought to be the case for gedunin (**6**) itself. However, the low *in vitro* activity of gedunin derivative **8** (IC_50_ = 90 μM) was attributed to the presence of a 6α-hydroxy group [20]. Herein, the *in vitro* anti-plasmodial activity of **5** was reported for the first time and was shown to be greater than that of the natural isolates **1**-**4** against the K1 strain of *P. falciparum* (IC_50_ = 5.0 μM). Thus, *in vitro* anti-plasmodial data from previous reports on **3**, **7** and **8** and data generated herein for 6α-acetoxygedunin (**3**) and 6α,7α-dihydroxy derivative **5** are consistent with the notion that within this group of gedunin derivatives, an *O*-acetyl group at the 6 and/or 7 position is not a requirement for significant *in vitro* anti-plasmodial activity against *P. falciparum*.

6α-acetoxygedunin (**3**) has been isolated previously from the flowers of *Carapa guianensis* and exhibited an IC_50_ = 2.8 μM against the FCR-3 strain of *P. falciparum* [20]. Herein, **3** was the most active of the isolates from RPSM and exhibited an IC_50_ = 7.0 μM against the K1 strain of *P. falciparum*. Limonoid **1** was half as active *in vitro* as **3**, in good agreement with previously published data and an indication of the modulation of *in vitro* activity caused by structural changes to ring D [20]. The *in vitro* activity of limonoid **3** and its availability on a larger scale by isolation from RPSM led us to further explore its anti-malarial potential in *P. berghei*-infected mice.

7-deacetoxy-7-oxogedunin (**4**) has been isolated previously from two species of Meliaceae, *Carapa guianensis* [20, 21] and *Pseudocedrela kotschyi* [37], and tested for *in vitro* anti-plasmodial activity against several strains of *P. falciparum*. In earlier studies, **4** exhibited *in vitro* activity against the K1 and FCR-3 strains of *P. falciparum* (IC_50_ = 2.5-4.1 μM) [20, 37]. However, herein, we found **4** to be relatively inactive against the K1 strain of *P. falciparum* (IC_50_ = 20.7 μM). Similarly, a previous report found **4** to be inactive against the W2 strain of *P. falciparum* (IC_50_ > 22.8 μM) [21]. Parasite strain specificity and/or variability of the anti-plasmodial response may be responsible for the mixed *in vitro* results obtained herein and elsewhere for **4**. We were thus induced to try to shed new light on the anti-malarial potential of **4** by further studying its activity in *P. berghei*-infected mice.

Herein, andirobin (**2**), a *seco*-gedunin, exhibited moderate *in vitro* anti-plasmodial activity (IC_50_ = 15.3 μM). In other work, a 7,8-*seco*-gedunin andirobin-like compound having an acetyl function instead of the furan ring and otherwise structurally analogous to **2**, as well as the 7,8-*seco*-gedunin, methyl angolensate, were found to have quite similar *in vitro* activities (IC_50_ = 12–15 μM) against the FCR-3 strain of *P. falciparum* [20]. Thus, an intact B ring may be important for optimal *in vitro* activity of gedunin derivatives against *P. falciparum*, and modification of the stereochemistry and type of substituent on the B ring at the 6 and 7 positions (*e.g*. of derivative **5**), could be an important strategy for optimizing the anti-plasmodial activity of gedunin derivatives.

Herein, 6α-acetoxygedunin (**3**) and 7-deacetoxy-7-oxogedunin (**4**) administered orally at doses of 50 and 100 mg/kg/day (Table 2), respectively, to *P. berghei* strain NK65-infected mice exhibited comparable *in vivo* anti-malarial activity (40% parasitemia suppression compared to untreated controls) to that reported previously by others for the natural isolate gedunin (**6**) at doses of 50 mg/kg/day in *P. berghei* strain ANKA-infected mice. Also, **6** reportedly did not suppress *P. berghei* in mice at oral doses of 25 or 100 mg/kg/day and thus did not exhibit dose–response [22]. However, herein, **3** administered orally at 100 mg/kg/day exhibited 66% suppression of parasitaemia of *P. berghei* on day 5 showing a dose–response pattern (and greater *in vivo* suppression of parasitemia than gedunin at this same dose). Comparable *in vivo* suppression of *P. berghei* to that of gedunin and clear dose–response highlight the potential of natural 6-substituted gedunin derivative **3**. While experience varies, substances exhibiting 30 to 40% *in vivo* parasitaemia suppression in mouse malaria models are considered to be moderately active whereas suppression ≥40% at the doses tested herein is associated with active anti-malarial compounds [38–40]. The present study was conducted with pure bred BALB/c mice infected with *P. berghei*, a reliable model that is similar to that used in the identification of drug candidates MK-4815, NITD609 and OZ439 that are now in clinical trials [41, 42].

Despite several studies on the *in vitro* anti-plasmodial activity of limonoids related to gedunin as discussed above, previous *in vivo* anti-malarial studies on gedunin derivatives were limited to gedunin (**6**) [22, 23, 43] and the semi-synthetic compound **10** [22]. The low oral activity of gedunin was attributed to low absorption and instability under physiologic conditions led to the semi-synthesis of deacetylgedunin (**9**). Compound **9** was unstable under physiologic conditions found in mice (and presumably in humans) [23]. For this reason, the 7-hydroxy group of compound **9** was methylated to provide 7-methoxy gedunin **10**. Compound **10** administered orally at doses of 50 mg/kg/day inhibited parasitaemia of *P. berghei* ANKA in mice by 68% [22] which is *in vivo* suppression of parasitaemia comparable to that observed herein for orally-administered isolate **3** at 100 mg/kg/day against *P. berghei* NK65 (Table 2).

It is important to keep in mind that highly potent and clinically useful anti-malarials were often discovered in plant materials or are derived from plant natural products used directly by humans as part of traditional practice. Often, however, clinically useful anti-malarials provide results that are not optimal for treatment of rodent malaria. Thus, quinine which was the basis for malaria treatment and prophylaxis in the 19th and early 20th centuries, exhibits a median effective dose (ED_50_) of 34 mg/kg/day in mice infected with *P. berghei* strain ANKA [44] and *Plasmodium vinckei*-infected mice exhibit recrudescence when treated with the artemisinin derivative sodium artesunate at doses <80 mg/kg/day [45]. Thus, the significant *in vivo* anti-malarial activity exhibited by limonoid natural product **3** has motivated to us to investigate the anti-malarial potential of its derivatives.

In the future, derivatives of **5** exhibiting *O*-alkyl and/or *O*-acyl groups in the 6 and 7 positions should be investigated. *O*-derivatization of the 7α-hydroxy group in **5** may be important for chemical stability and *in vivo* anti-malarial activity given the structural analogy of **5** with 7-deacetyl gedunin (**9**) and the low *in vivo* anti-malarial activity of the latter compound. The low stability of **9** under simulated gastric (low pH) conditions was shown to be due to B ring-opening provoked by the intrinsic reactivity of the 7α-hydroxy moiety [23]. Thus, *O*-alkylation or *O*-acylation with a sterically hindered ester group on the 7α-hydroxy (and 6α-hydroxy) group of **5** should be investigated as a means to generate novel substances for *in vitro* and *in vivo* study of anti-malarial activity and could lead to a better understanding of the effects of B ring substituents on the stability and anti-malarial activity of gedunin derivatives.

## Conclusions

Today, in the fight against malaria, the clinically most relevant anti-malarials, synthetic quinolines and semi-synthetic artemisinin derivatives, owe their origins to the natural products quinine and artemisinin, respectively, found in traditionally-used plants. Furthermore, medicinal plants selected through the ethnopharmacologic approach are proving to be important sources of drugs introduced in the past decades. Thus, limonoids **1**-**4** were isolated from *C. guianensis* waste seed materials and their *in vitro* efficacy against *P. falciparum* and cytotoxicity to normal cells was evaluated. Limonoids **3** and **4** were further evaluated *in vivo* against *P. berghei* in mice. Our *in vitro* and *in vivo* results were contextualized within what is known on the inhibitory activity of gedunin derivatives from previous studies. 6α-acetoxygedunin (**3**) was shown herein to exhibit significant *in vivo* anti-malarial activity and dose–response that make this natural derivative of gedunin a rival to gedunin (**6**) itself as a model structure for further investigation. Future work will focus on the *in vitro* and *in vivo* activity of 6 and 7-substituted derivatives of semi-synthetic limonoid **5**. Also, as a means to avoid monotherapy and to enhance *in vivo* anti-malarial activity, studies are planned on the potential synergism of **3** and derivatives of **5** with dillapiole, a natural product known to strongly inhibit cytochrome P450 and significantly enhance the *in vivo* anti-malarial activity of gedunin (**6**) and gedunin derivative **10** according to a previous study in a mouse malaria model [22].

## Electronic supplementary material

Additional file 1: **Spectral data for isolated limonoids 1-4 and prepared derivative 5;** ^**1**^ **H and** ^**13**^ **C NMR and HRMS data for these compounds are presented in this file.** (DOCX 22 KB)

## Abbreviations

ACT: Artemisinin-based combined therapy
CC: Column chromatography
CQR: Chloroquine-resistant
DMEM: Dulbecco’s Modified Eagle’s Medium
DMSO: Dimethyl sulfoxide
ED_50_: Median effective dose
HPLC: High performance liquid chromatography
HRMS: High resolution mass spectrometry
IC_50_: Median inhibition concentration of growth
INPA: National Institute for Amazon Research
NMR: Nuclear magnetic resonance
RPMI: Roswell Park Memorial Institute (cell culture medium)
RPSM: Residual pressed seed material
TLC: Thin-layer chromatography.

## References

[1] WHO (2012). World malaria report 2012.

[2] Marques MM, Costa MRF, Santana Filho FS, Vieira JLF, Nascimento MTS, Brasil LW, Nogueira F, Silveira H, Reyes-Lecca RB, Monteiro WM, Lacerda MVG, Alecrim MGC (2013). *Plasmodium vivax* chloroquine resistance and anemia in the western Brazilian Amazon. Antimicrob Agents Chemother.

[3] Cruz LR, Spangenberg T, Lacerda MVG, Wells TNC (2013). Malaria in South America: a drug discovery perspective. Malar J.

[4] Gama BE, Lacerda MVG, Daniel-Ribeiro CT, Ferreira-da-Cruz MFF (2011). Chemoresistance of *Plasmodium falciparum* and *Plasmodium vivax* parasites in Brazil: consequences on disease morbidity and control. Mem Inst Oswaldo Cruz.

[5] Newman DJ, Cragg GM (2012). Natural products as sources of new drugs over the 30 years from 1981 to 2010. J Nat Prod.

[6] Guantai E, Chibale K (2011). How can natural products serve as a viable source of lead compounds for the development of new/novel anti-malarials?. Malar J.

[7] Ginsburg H, Deharo E (2011). A call for using natural compounds in the development of new antimalarial treatments–an introduction. Malar J.

[8] Schmidt TJ, Khalid SA, Romanha AJ, Alves TMA, Biavatti MW, Brun R, Costa FB, Castro SL, Ferreira VF, Lacerda MVG, Lago JHG, Leon LL, Lopes N, Amorim RCN, Niehues M, Ogungbe IV, Pohlit AM, Scotti MT, Setzer WN, Soeiro MNC, Steindel M, Tempone MG (2012). The potential of secondary metabolites from plants as drugs or leads against protozoan neglected diseases – part I. Curr Med Chem.

[9] Schmidt TJ, Khalid SA, Romanha AJ, Alves TMA, Biavatti MW, Brun R, Costa FB, Castro SL, Ferreira VF, Lacerda MVG, Lago JHG, Leon LL, Lopes N, Amorim RCN, Niehues M, Ogungbe IV, Pohlit AM, Scotti MT, Setzer WN, Soeiro MNC, Steindel M, Tempone MG (2012). The potential of secondary metabolites from plants as drugs or leads against protozoan neglected diseases - part II. Curr Med Chem.

[10] Achan J, Talisuna AO, Erhart A, Yeka A, Tibenderana JK, Baliraine FN, Rosenthal PJ, D’Alessandro U (2011). Quinine, an old antimalarial drug in a modern world: role in the treatment of malaria. Malar J.

[11] Pohlit AM, Lima RBS, Frausin G, Silva LF R e, Lopes SC, Moraes CB, Cravo P, Lacerda MVG, Siqueira AM, Freitas-Junior LH, Costa FTM (2013). Amazonian plant natural products: perspectives for discovery of new antimalarial drug leads. Molecules.

[12] Ferraz IDK, Camargo JLC, Sampaio PTB (2002). Sementes e plântulas de andiroba (*Carapa guianensis* Aubl. e *Carapa procera* DC.): aspecto botânico, ecológico e tecnológico. Acta Amazon.

[13] Mendonça AP, Ferraz IDK (2007). Óleo de andiroba: processo tradicional da extração, uso e aspectos sociais no estado do Amazonas, Brasil. Acta Amazon.

[14] Costa-Silva JH, Lima CR, Silva EJR, Araújo AV, Franga MCCA, Ribeiro AR, Arruda AC, Lafayette SSL, Walderley AG (2007). A toxicological evaluation of the effect of *Carapa guianensis* Aubl. on pregnant Wistar rats. J Ethnopharmacol.

[15] Ambrozin ARP, Leite AC, Bueno FC, Vieira PC, Fernandes JB, Bueno OC, Pagnocca FC (2006). Limonoids from andiroba oil and *Cedrela fissilis* and their insecticidal activity. J Braz Chem Soc.

[16] Mendonça FAC, Silva KFS, Santos KK, Ribeiro Júnior KAL, Sant’Ana AEG (2005). Activities of some Brazilian plants against larvae of the mosquito *Aedes aegypti*. Fitoterapia.

[17] Penido C, Costa KA, Costa MFS, Pereira JFG, Siani AC, Henrique MGMO (2006). Inhibition of allergen-induced eosinophil recruitment by natural tetranortriterpenoids is mediated by the suppression of IL-5, CCL11/eotaxin and NFnB activation. Int Immunopharmacol.

[18] Klimas CA, Kainer KA, Wadt LHO (2007). Population structure of *Carapa guianensis* in two forest types in the southwestern Brazilian Amazon. For Ecol Manag.

[19] Miranda Junior RN, Dolabela MF, Silva MN, Póvoa MM, Maia JGS (2012). Antiplasmodial activity of the andiroba (*Carapa guianensis* Aubl., Meliaceae) oil and its limonoid-rich fraction. J Ethnopharmacol.

[20] Tanaka Y, Sakamoto A, Inoue T, Yamada T, Kikuchi T, Kajimoto T, Muraoka O, Sato A, Wataya Y, Kim H, Tanaka R (2012). Andirolides H-P from the flower of andiroba (*Carapa guianensis*, Meliaceae). Tetrahedron.

[21] Mackinnon S, Dust T, Arnason JT (1997). Antimalarial activity of tropical Meliaceae extracts and gedunin derivatives. J Nat Prod.

[22] Omar S, Godard K, Ingham A, Hussan H, Wongpanich V, Pezzuto J, Durst T, Eklu C, Gbeassor M, Sanchez-Vidas P, Poveda I, Philogene BJR, Arnason JT (2003). Antimalarial activities of gedunin and 7-methoxygedunin and synergistic activity with dillapiol. Ann Appl Biol.

[23] Omar S, Zhang J, MacKinnon S, Leaman D, Durst T, Philogene BJL, Arnason JT, Sanchez-Vindas PE, Poveda L, Tamez PA, Pezzuto JM (2003). Traditionally-used antimalarials from the Meliaceae. Curr Top Med Chem.

[24] Onguéné PA, Ntie-Kang F, Lifongo L, Ndom J, Sippl W, Mbaze L (2013). The potential of anti-malarial compounds derived from African medicinal plants. Part I: A pharmacological evaluation of alkaloids and terpenoids. Malar J.

[25] *The Species Link Project*. [http://www.splink.org.br/index?lang=en

[26] Trager W, Jensen JB (1976). Human malaria parasites in continuous culture. Science.

[27] Silva LF R e, Montoia A, Amorim RCN, Melo MR, Henrique MC, Nunomura SM, Costa MRF, Andrade Neto VF, Costa DS, Dantas G, Lavrado J, Moreira R, Paulo A, Pinto AC, Tadei WP, Zacardi RS, Eberlin MN, Pohlit AM (2012). Comparative *in vitro* and *in vivo* antimalarial activity of the indole alkaloids ellipticine, olivacine, cryptolepine and a synthetic cryptolepine analog. Phytomedicine.

[28] Peters W (1965). Drug resistance in *Plasmodium berghei* Vincke and Lips, 1948. I. Chloroquine resistance. Exp Parasitol.

[29] Silva LF R e, Lima ES, Vasconcellos MC, Aranha ESP, Costa DS, Santos EVM, Morais SKR, Alecrim MGC, Nunomura SM, Struwe L, Andrade Neto VF, Pohlit AM (2013). *In vitro* and *in vivo* antimalarial activity and cytotoxicity of extracts, fractions and a substance isolated from the Amazonian plant *Tachia grandiflora* (Gentianaceae). Mem Inst Oswaldo Cruz.

[30] Ahmed SA, Gogal RM, Walsh JE (1994). A new rapid and simple non-radioactive assay to monitor and determine the proliferation of lymphocytes: an alternative to [^3^H] thymidine incorporation assay. J Immunol Methods.

[31] Lima ES, Pinto ACS, Nogueira KL, Silva LF R e, Almeida PDO, Vasconcellos MC, Chaves SCM, Tadei WP, Pohlit AM (2013). Stability and antioxidant activity of semi-synthetic derivatives of 4-nerolidylcatechol. Molecules.

[32] Kraus W, Cramer R (1978). 17-epi-azadiradion und 17-β-hydroxy-azadiradion, zwei neue inhaltsstoffe aus *Azadirachta indica* A. Juss Tetrahedron Lett.

[33] Jittaniyom C, Sommit D, Muangsin N, Pudhom K (2012). Andirobin from *X. moluccensis*. Acta Crystallogr Sect E: Struct Rep Online.

[34] Hofer M, Greger H, Mereiter M (2009). 6α-acetoxygedunin. Acta Crystallogr Sect E: Struct Rep Online.

[35] Silva SG, Nunomura RCS, Nunomura SM (2012). Limonoides isolados dos frutos de *Carapa guianensis* Aublet (Meliaceae). Quim Nova.

[36] Lavie D, Levy EC, Zelnik R (1972). The constituents of *Carapa guianensis* Aubl. and their biogenetic relationship. Bioorg Chem.

[37] Hay AE, Loset JH, Ahua KM, Diallo D, Brun R, Hostettmann K (2007). Limonoid orthoacetates and antiprotozoal compounds from the roots of *Pseudocedrela kotschyi*. J Nat Prod.

[38] Andrade AA, Varotti FP, Freitas IO, Souza MV, Vasconcelos TR, Boechat N, Krettli AU (2007). Enhanced activity of mefloquine and artesunic acid against *Plasmodium falciparum in vitro* and *P. berghei* in mice by combination with ciprofloxacin. Eur J Pharmacol.

[39] Krettli AU, Adebayo JO, Krettli LG (2009). Testing of natural products and synthetic molecules aiming at new antimalarials. Curr Drug Targets.

[40] Coutinho JP, Aguiar AC, Lima JC, Rocha MG, Zani CL, Alves TM, Santana AE, Pereira MM, Krettli AU (2013). *Aspidosperma* (Apocynaceae) plant cytotoxicity and activity toward malaria parasites. Part I: *Aspidosperma nitidum* (Benth.) used as a remedy to treat fever and malaria in the Amazon. Mem Inst Oswaldo Cruz.

[41] Aguia AC, Rocha EM, Souza NB, França TC, Krettli AU (2012). New approaches in antimalarial drug discovery and development: a review. Mem Inst Oswaldo Cruz.

[42] Jiménes-Díaz MB, Viera S, Fernández-Alvaro E, Angulo-Barturen I (2014). Animal models of efficacy to accelerate drug discovery in malaria. Parasitology.

[43] Bray DH, Warhust DC, Connolly JD, O’Neil MJ, Phillipson JD (1990). Plants as sources of antimalarial drugs*.* Part 7. Activity of some species of Meliaceae plants and their constituent limonoids. Phytother Res.

[44] Garavito G, Bertani S, Quiliano M, Valentin A, Aldana I, Deharo E (2012). The *in vivo* antimalarial activity of methylene blue combined with pyrimethamine, chloroquine and quinine. Mem Inst Oswaldo Cruz.

[45] Lombard MC, N’Da DD, Tran Van Ba C, Wein S, Norman J, Wiesner L, Vial H (2013). Potent *in vivo* anti-malarial activity and representative snapshot pharmacokinetic evaluation of artemisinin-quinoline hybrids. Malar J.

