# Supplementary material for: In vitro and in vivo anti-malarial activity of limonoids isolated from the residual seed biomass from Carapa guianensis (andiroba) oil production
Source: Malar J. 2014 Aug 13;13:317. doi: 10.1186/1475-2875-13-317 (PMC4138406; doi:10.1186/1475-2875-13-317)
Supplement: Supplementary file 1 — Additional file 1: Spectral data for isolated limonoids 1-4 and prepared derivative 5; 1 H and 13 C NMR and HRMS data for these compounds are presented in this file. (DOCX 22 KB) [file 12936_2014_3350_MOESM1_ESM.docx]

**Additional File 1**

**Spectral Data for isolated limonoids 1-4 and prepared derivative 5**

Compounds **1**-**5** were in general characterized by high resolution mass spectra (HRMS, Waters, Xevo Model) obtained by direct infusion of each pure compound dissolved in methanol/water. Nuclear magnetic resonance (NMR) spectra were obtained of pure samples on a Varian Inova 500 MHz and Bruker Avance, 600 MHz and 400 MHz. HRMS and full ^1^H and ^13^C NMR data are provided below.

6α-acetoxyepoxyazadiradione (**1**): crystalline solid, molecular formula: C_30_H_36_O_8_. HRMS: *m/z* 547.2324 ([M+Na]^+^, calcd. exact mass = *m/z* 547.2308, Δ = 2.9 ppm). ^1^H NMR (500 MHz, CDCl_3_) δ: 7.43 (*t*, 1H, *J* = 1.6 Hz, H21), 7.41 (*bs*, 1H, H23), 7.16 (*d*, 1H, *J* = 10.1 Hz, H1), 6.26 (*d*, 1H, *J* = 1.6 Hz, H22), 5.99 (*d*, 1H, *J* = 10.1 Hz, H2), 5.40 (*dd*, 1H, *J* = 12.5, 2.6 Hz, H6), 5.06 (*d*, 1H, *J* = 2.6 Hz, H7), 3.92 (*s*, 1H, H17), 3.46 (*s*, 1H, H15), 2.71 (*dd*, 1H, *J* = 12.5, 4.0 Hz, H9), 2.54 (*d*, 1H, *J* = 12.5 Hz, H5), 2.19, 1.88, 2.03, 1.95 (*m*, 1H each, H11 and H12), 2.11 (*s*, 3H, 6-CO_2_CH_3_), 2.05 (*s*, 3H, 7-CO_2_CH_3_),1.36 (*s*, 3H, H30), 1.29 (*s*, 3H, H28) 1.24 (*s*, 3H, H19), 1.20 (*s*, 3H, H29), 1.07 (*s*, 3H, H18). ^13^C NMR (125 MHz, CDCl_3_) δ: 207.9 (C16), 204.4 (C3), 169.9 (C6-CO_2_CH_3_), 169.8 (C7-CO_2_CH_3_), 156.7 (C1), 142.5 (C21), 141.6 (C23), 126.5 (C2), 116.4 (C20), 110.9 (C22), 73.0 (C7), 72.2 (C14), 69.8 (C6), 57.0 (C15), 50.8 (C17), 48.5 (C5), 45.2 (C4), 43.2 (C8), 42.5 (C13), 40.5 (C10), 38.4 (C9), 31.6 (C28), 28.6 (C12), 24.7 (C18), 21.5 (C19), 21.2 (C6-CO_2_CH_3_), 21.1 (C7-CO_2_CH_3_), 20.2 (C29), 19.0 (C30),16.1 (C11).

Andirobin (**2**): crystalline solid molecular, formula: C_27_H_32_O_7_. HRMS: *m/z* 491.2023 ([M+Na]^+^, calcd. exact mass= *m/z* 491.2046, Δ= 4.7 ppm). ^1^H NMR (500 MHz, CDCl_3_) δ: 7.44 (*t*, 1H, *J* = 1.6 Hz, H23), 7.43 (*d*, 1H, *J* = 1.0 Hz, H21), 7.17 (*d*, 1H, *J* = 10.5 Hz, H1), 6.36 (*dd*, 1H, *J* = 1.6 and 1.0 Hz, H22), 6.09 (*d*, 1H, *J* = 10.5 Hz, H2), 5.5 (*s*, 1H, H17), 5.40 (*s*, 1H, H30), 5.29 (*s*, 1H, H30) 4.07(*s*, 1H, H15), 3.73 (*s*, 3H, 7-OCH_3_), 2.71 (*dd*, 1H, *J* = 7.3 Hz, H5), 2.02 (*dt*, *J* = 14.0, 3.0, 3.0 Hz, H11α), 2.49 (*d,* 1H, *J* = 7.0 Hz, H9), 1.82 (*m*, 1H, H11), 1.68 (*dd*, 1H, *J* = 14.0, 5.0 Hz, H12), 1.26 (*m,*1H, H12), 1.14 (*s*, 3H, H28), 1.11 (*s*, 3H, H29), 1.01 (*s*, 3H, H19), 0.96 (*s*, 3H, H18). ^13^C NMR (125 MHz, CDCl_3_) δ: 203.8 (C3), 174.4 (C7), 166.8 (C16), 153.5 (C1), 143.3 (C23), 140.9 (C21), 138.9 (C8), 125.7 (C2), 122.4 (C30), 119.7 (C20), 109.7 (C22), 77.4 (C17), 67.9 (C14), 55.4 (C15), 52.2 (C7-OCH_3_), 48.7 (C9), 46.1 (C4), 43.0 (C10), 42.8 (C5), 38.6 (C13), 31.5 (C6), 29.5 (C12), 22.7 (C29), 22.5 (C28), 21.2 (C11), 20.2 (C19), 14.6 (C18).

6α-acetoxygedunin (**3**):crystalline solid, molecular formula C_30_H_36_O_9_. HRMS: *m/z* 541.2426 ([M+H]^+^, calcd.exact mass = *m*/*z* 541.2438, Δ = 2.2 ppm).^1^H NMR(400 MHz, CDCl_3_) δ: 7.50 (*bd*, 1H, *J* = 1.3 Hz, H21), 7.40 (*bd*, 1H, *J* = 1.3 Hz, H23), 7.10 (*d*, 1H, *J* = 10.1 Hz, H1), 6.36 (*t*, 1H, *J =* 1.32 Hz, H22), 5.98 (*d*, 1H, *J* = 10.1 Hz, H2), 5.64 (*s*, 1H, H17), 5.31 (*dd,*1H, *J* = 12.5, 2.4 Hz, H6), 4.93 (*d,*1H, *J* = 2.4 Hz, H7), 3.65 (*s*, 1H, H15), 2.57 (*m*, 1H, H9), 2.56 (*d,*1H, *J* = 12.5 Hz, H5), 2.18 (*s,* 3H, 7-OAc), 2.07 (*s*, 3H, 6-OAc), 2.02, 2.57, 1.77 and 1.63 (*m*, 1H each, H11 and H12), 1.30 (*s,* 3H, H30), 1.29 (*s,* 3H, H29), 1.28 (*s,* 3H, H18), 1.25 (*s,* 3H, H19), 1.20 (*s,* 3H, H29). ^13^C NMR (125 MHz, CDCl_3_) δ: 204.1 (C3), 170.1 and 170.0 (6-COCH_3_ and 7-COCH_3_), 167.1 (C16), 156.2 (C1), 143.1 (C23), 141.2 (C21), 126.6 (C2), 120.3 (C20), 109.8 (C22), 78.1 (C17), 72.6 (C7), 69.6 (C6), 69.5 (C14), 56.2 (C15), 47.4 (C5), 44.9 (C4), 43.1 (C8), 40.6 (C10), 38.8 (C13), 38.4 (C9), 31.6 (C28), 25.9 (C12), 20.9 (C19), 21.4 (7-COCH_3_), 21.2 (6-COCH_3_), 20.2 (29), 17.9 (C18), 15.0 (C11).

7-deacetoxy-7-oxogedunin (**4**): crystalline solid, molecular formula: C_26_H_30_O_6_. HRMS: *m/z* 439.2149 ([M+H]^+^, calcd. exact mass *m/z* 439.2121, Δ = 6.4 ppm).^1^H NMR (600 MHz, CDCl_3_) δ: 7.44 (*t*, 1H, *J* = 0.7 Hz, H21), 7.41 (*t*, 1H, *J* = 1.7 Hz, H23), 7.13 (*d*, 1H, *J* = 10.2 Hz, H1), 6.38 (*dd*, 1H, *J* = 1.7, 0.7 Hz, H22), 5.95 (*d,* 1H, *J* = 10.2 Hz, H2), 5.5 (*s*, 1H, H17), 3.9 (*s,* 1H, H15), 2.94 (*t*, 1H, *J* = 14.3 Hz, H6β), 2.43 (*dd*, 1H, *J* = 14.3, 3.0 Hz, H6α), 2.23 (*dd*, 1H, *J* = 11.9, 1.5 Hz, H9), 2.21 (*dd*, 1H, *J* = 14.3, 3.0 Hz, H5), 2.01, 1.87, 1.90, 1.50 (*m*, 1H each, H11 and H12) 1.38 (*s*, 3H, H19), 1.24 (*s,* 3H, H30), 1.18 (*s*, 3H, H18), 1.16 (*s,* 3H, H29), 1.15 (*s,* 3H, H28). ^13^C NMR (125 MHz, CDCl_3_) δ: 208.2 (C7), 203.3 (C3), 167.2 (C16), 156.0 (C1), 143.2 (C23), 141.0 (C21), 126.5 (C2), 120.2 (C20), 109.9 (C22), 78.0 (C17), 65.2 (C14), 54.6 (C5), 53.6 (C15), 53.2 (C8), 47.6 (C9), 45.3 (C4), 39.6 (C10), 37.8 (C13), 36.8 (C6), 32.2 (C12), 27.0 (C28), 20.9 (C29), 20.7 (C18), 19.9 (C19), 17.4 (C30), 17.2 (C11).

6α-hydroxydeacetylgedunin (**5**): white solid, molecular formula: C_26_H_32_O_7_. HRMS: *m/z* 457.2139 ([M+H]^+^, calcd. exact mass = *m/z* 457.2226). ^1^H NMR (400 MHz, CDCl_3_) δ: 7.45 (*bd*, 1H, *J* = 0.6 Hz, H21), 7.44 (*t,* 1H, *J* = 1.7 Hz, H23), 7.08 (*d*, 1H, *J* = 10.1 Hz, H1), 6.36 (*dd*, 1H, *J* = 1.7, 0.6 Hz, H22), 5.93 (*d*, 1H, *J* = 10.1 Hz, H2), 5.64 (*s,*1H, H17), 4.17 (*dd*, 1H, *J* = 11.6, 2.4 Hz, H6), 3.97 (*s,* 1H, H15), 3.39 (*d*, 1H, *J* = 2.4 Hz, H7), 2.57 (*dd,* 1H, *J* = 12.4 and 6.0 Hz, H9),2.42 (*d*, 1H, *J* = 11.6 Hz, H5), 1.98, 1.78, 1.75, 1.60 (*m,*1H each, H11 and H12),1.42 (*s*, 3H, H29), 1.37 (*s,* 3H, H28), 1.30 (*s*, 3H, H18), 1.25 (*s*, 3H, H19) 1.13 (*s*, 3H, H30). ^13^C NMR (125 MHz, CDCl_3_) δ: 205.6 (C3), 168.3 (C16), 156.9 (C1), 143.0 (C23), 141.2 (C21), 126.4 (C2), 120.6 (C20), 109.4 (C22), 78.6 (C17), 74.9 (C7), 69.9 (C14), 69.0 (C6), 57.8 (C15), 48.4 (C5), 45.4 (C4), 43.4 (C8), 40.4 (C10), 38.3 (C13), 36.7 (C9), 32.1 (C28), 26.4 (C12), 21.7 (C19), 20.5 (C29), 17.9 (C18), 15.2 (C11), 15.1 (C30).
